# Skin Diseases and Syphilis

**Published:** 1895-03

**Authors:** 


### SKIN DISEASES AND SYPHILIS.

EDITED BY DR. M. B. HUTCHINS.

### ON THE NATURE OF ECZEMA.

DR. ERNST SCHWIMMER, Budapest. (*Wiener Medizinische Wochenschrift*, No. 30–34, 1894.)

"What we know of eczema at the present time has reference to anatomical changes and clinical appearances." The anatomical changes consist in a catarrhal inflammation, the essential pathological processes being those of "hyperemia and transudation." From a clinical point of view, the disease presents a characteristic eruption, accompanied by itching, and running a definite course, and occurs only in persons with a certain individual disposition. Eczema may thus be defined as "an inflammatory affection of the upper corium and neighboring malphigian layer, which, with the accompanying exudation, leads to an eruption, together with an irritable condition of the cutaneous nerves and a more or less lasting nutritional disturbance of the affected tissues."

In discussing the etiology of eczema, Dr. Schwimmer groups the possible causes under the heads of (1) local and (2) general, subdividing the latter into (*a*) constitutional diseases and diseases of internal organs, (*b*) causes connected with the nervous system, (*c*) micro-organisms. The local causes afford no explanation of the etiology beyond the fact that local irritation can only produce true eczema in individuals predisposed. That eczema has any real relation with general constitutional conditions or with diseases of internal organs the author doubts. That it is dependent upon the action of micro-organisms he disputes, and considers any such connection altogether unproved. He shows that there is much more evidence, clinical and pathological, for considering nervous influence as the chief factor in the etiology of eczema. He suggests, moreover, that the development of the skin and nervous system from the same embryonic layer tends to point also in this direction. Concerning the etiology of eczema he concludes as follows: "The morbid agent of the eczema eruption is unknown to us; it appears to lie in the skin itself, and possibly consists in a developmental defect in the nervous-tissue element."—H. G. ADAMSON, in *British Journal of Dermatology*, January, 1895.

---

### THE EFFECT OF EARLY ANTI-SYPHILITIC TREATMENT ON THE NERVOUS SYSTEM.

ARTHUR DEUTSCH. (*Archiv. fur Dermatologie und Syphilis*, November, 1894.)

Dr. Deutsch reviews the opinions of various authors for and against the early treatment of syphilis, and gives the results of a research undertaken to determine the effect of such treatment in relation to nervous lesions produced by syphilis. In discussing the statistics of the opponents of early treatment he shows that in those cases in which nervous lesions had developed at a later period the treatment had not been commenced sufficiently early. He denies that the severe nervous symptoms in these cases are the result of anti-syphilitic treatment. He describes the early nervous symptoms which may occur in untreated cases, even before the appearance of secondary eruptions, and which, depending upon a hyperemic condition of the brain and spinal cord, produced by the syphilitic virus, predispose to later organic lesions. From observations on a large number of cases he shows that, if treatment be begun immediately after contagion, this hyperemic condition, with resulting nervous symptoms, is prevented, and the tendency to later organic lesions is greatly diminished, while, should the treatment be delayed till the appearance of secondary symptoms, and until after the development of the hyperemic condition of the brain and cord, later organic nervous lesions are much more likely to occur.—H. G. ADAMSON, in *British Journal of Dermatology*, January, 1895.

---

### CUTANEOUS VERRUCOSE TUBERCULOSIS FROM ACCIDENTAL INOCULATION, IN THE ATTENDANT ON A PHTHISICAL PERSON.

Excision and igni-puncture. DR. THIBAUDET (DE ST. CLAUDE). (*Journal des Sciences Médicales de Lille*, October 20, 1894.)

The case published by Dr. Thibaudet is interesting in that it shows distinctly its origin by local inoculation. The patient, a woman, aged forty, had slightly wounded the back of her hand while making the bed for her phthisical son. The wound failed to heal, and little by little developed into a warty and scaly plaque, which, when the patient first came under observation eighteen months later, was of the dimensions of a two-franc piece. Tubercle bacilli were found in an excised portion. Treatment by excision of the whole area of disease was selected in preference to igni-puncture or scarification, for the following reasons: The disease was limited to one spot, and in a situation favorable for union by first intention, even after extensive removal of tissue, and, moreover, a rapid cure was advisable on account of the interference of the lesion with the patient's occupation of dressmaker.

Dr. Thibaudet points out that the *verrucose* type is that usually observed in "professional inoculations—*i. e.*, in doctors, nurses, veterinary surgeons, butchers, etc.—and suggests as a prophylactic that persons thus exposed to local contamination should watch with great care the integrity of the skin, and in case of any abrasion to immediately cover such with an antiseptic collodion or similar protection.—H. G. ADAMSON, in *British Journal of Dermatology*, January, 1895.

---

### COAL-TAR IN SKIN DISEASES.

Dr. L. Leistikow (in *La Semaine Médicale*, No. 59, 1894) advocates the use of coal-tar in the treatment of pruritus and other itching affections, and states that he has found it produce more lasting effects than wood-tar, oil of cade, etc. It has proved especially valuable in the treatment of dry eczemas of the scalp, neck, abdomen, genitals, and extremities, in prurigo, in trichophytiasis, and in psoriasis of the hairy scalp, knees, and elbows. Its use should be restricted to localized skin lesions, lest symptoms of poisoning result; it is of itself liable to give rise to an intense erythema, and hence should not be applied to the face, nor should it be used unless the patient can give up his occupation during treatment.

The following preparation is used, application being made by means of a camel's-hair brush:

| | |
|---|---|
| Coal-tar | ℥ iss. |
| Alcohol (95 per cent.) | ℥ i. |
| Ether | ℥ sss. |

The tar may be easily removed by rubbing with olive oil.—*Brit. Journal Dermatology*, January, 1895.

---

### THE TREATMENT OF PRURITUS VULVÆ. (*Journal de Médicine de Paris*, No. 25, 1894.)

It is recommended that in pruritus of diabetic origin the diet should be regulated, specific treatment adopted, and very hot water applied locally.

When it depends on a local condition, such as chronic eczema of the genital organs, a gonorrheal vaginitis, a chronic vaginitis with leucorrheal discharge or vaginismus with hysterical symptoms, the following application should be employed locally three times daily after irrigation of the vagina with three or four pints of warm permanganate of potash solution (1 : 1000).

| | | |
|---|---|---|
| ℞ | Water | 450 parts. |
| | Alcohol (90 per cent.) | 50 " |
| | Mercuric chloride | 1 " |
| | Indigo carmine | .05 " |

When the burning and itching are severe, washing with hot water proves efficacious. Salves and ointments should be avoided, lest by their fermentation they increase the irritation. No stimulating drinks or foods should be allowed.—*Brit. Journal Dermatology*, January, 1895.

---

### FRECKLES AND CLOASMA.

In the *Wiener Medizinische Presse*, No. 43, 1894, the treatment of freckles and cloasma is discussed. For freckles the application of the following preparation night and morning is recommended:

| | |
|---|---|
| Corosive sublimate | gr. iv to xv. |
| Colorless extract hamamelis | āā ℥ iss. |
| Glycerine | |
| Alcohol | |

As soon as pronounced irritation of the skin has been produced the remedy should be discontinued until after the symptoms of irritation have disappeared under treatment by zinc ointment, when it should be again resumed. If the above application be found too irritating—

| | |
|---|---|
| Hydrochlorate of cocaine | gr. xv. |
| Boric acid | ʒ iv. |
| Rectified alcohol | ℥ vi. ʒ ii. |

may be applied on compresses with almost as good result.

| | |
|---|---|
| In Cloasma—Corrosive sublimate | gr. ix. |
| Ammonium chloride | ʒ i. |
| Rectified alcohol | ℥ iss. |
| Colorless extr. hamamelis | ℥ iii. |

has been used locally with marked success.—*Brit. Journal Dermatology*, January, 1895.

TREATMENT OF CANCER OF THE FACE. (*Universal Med. Journal*, August, 1894.)

M. A. Darier reports a series of cases of this affection treated with marked success by daily application of 1 to 20 methyl-blue. If properly used, he states, it may lead to cure without any associated treatment, but it is more rapid in its action if the affected area be first touched with the galvano-cautery. When the tumor is deeply seated it is his custom to inject the drug hypodermically. He believes it to have a specific action upon the disease. When the new growth has caused destruction of a large area of the skin, grafting during the third week is recommended.—*Brit. Journal Dermatology*, January, 1895.

---

PILOCARPINE IN URTICARIA. (*Med. Record.*)

Dr. Abrams, of New York, recommends very highly the use of pilocarpine in the treatment of acute and chronic urticaria. If the drug be administered with care, and the dose increased gradually, there is no danger in its use. To adults it should be administered hypodermically in doses of $\frac{1}{6}$ to $\frac{1}{2}$ a grain. Children of one year may be treated with $\frac{1}{20}$ to $\frac{1}{8}$ grain by the mouth every evening. Children three years old take from $\frac{1}{15}$ to $\frac{1}{6}$ of a grain of this drug.—*Brit. Journal Dermatology*, January, 1895.

---

DANGERS OF BETA-NAPHTHOL. (*La Semaine Médicale*, No. 59, 1894.)

Dr. Baatz points out a possible danger attending the use of beta-naphthol as an ointment. Two children, aged eight and six years, were treated for scabies with a salve containing two per cent. of this drug. Three weeks later, when the skin affection was cured, albuminuria and œdema of both legs appeared, and one child died. Neither of the children had suffered from symptoms of renal disease before. A *post mortem* examination was obtained, and the diagnosis confirmed.--*Brit. Journal Dermatology*, January, 1895.

